# Nomogram for predicting prognosis of patients with metastatic melanoma after immunotherapy: A Chinese population–based analysis

**DOI:** 10.3389/fimmu.2022.1083840

**Published:** 2022-12-22

**Authors:** Jingjing Zhao, Dandan Li, Songzuo Xie, Xinpei Deng, Xizhi Wen, Jingjing Li, Zhengrong Wu, Xinyi Yang, Minxing Li, Yan Tang, Xiaoshi Zhang, Ya Ding

**Affiliations:** ^1^ Collaborative Innovation Center for Cancer Medicine, State Key Laboratory of Oncology in South China, Sun Yat-Sen University Cancer Center, Guangzhou, China; ^2^ Department of Biotherapy, Sun Yat-Sen University Cancer Center, Guangzhou, China; ^3^ Department of Pathology, School of Basic Medical Sciences, Southern Medical University, Guangzhou, China

**Keywords:** metastatic melanoma, immunotherapy, nomogram, anti-pd-1 treatment, baseline indicators

## Abstract

**Background:**

Previous studies indicated the evidence that baseline levels of thyroid antibodies, thyroid status, and serum lactate dehydrogenase (LDH) and M stage may influence the prognosis of patients with advanced or metastatic melanoma treated with immune checkpoint inhibitors that targets programmed cell death-1 (PD-1) or programmed death ligand 1, which reported that dramatic improvements in survival rates were observed; however, the presence of controversy has prevented consensus from being reached. Study objectives were to develop a nomogram to identify several prognostic factors in Chinese patients with metastatic melanoma receiving immunotherapy.

**Methods:**

This retrospective study included 231 patients from Sun Yat-sen University Cancer Center, and patients were split into internal cohort (n = 165) and external validation cohort (n = 66). We developed a nomogram for the prediction of response and prognosis on the basis of the levels of serum thyroid peroxidase antibody (A-TPO), free T3 (FT3), and LDH and M stage that were measured at the baseline of anti–PD-1 infusion. In addition, the follow-up lasted at least until 5 years after the treatment or mortality. RECIST v1.1 was used to classify treatment responses.

**Results:**

Chi-square test showed that PD-1 antibody was more effective in patients with melanoma with high level baseline FT4 or earlier M stage. A multivariate Cox analysis showed that baseline FT3 (P = 0.009), baseline A-TPO (P = 0.016), and LDH (P = 0.013) levels and M stage (P < 0.001) independently predicted overall survival (OS) in patients with melanoma. The above factors are integrated, and a prediction model is established, i.e., nomogram. Survival probability area-under-the-curve values of 1, 2, and 3 years in the training, internal validation, and external validation cohorts showed the prognostic accuracy and clinical applicability of nomogram (training: 0.714, 0.757, and 0.764; internal validation: 0.7171963, 0.756549, and 0.7651486; external validation: 0.748, 0.710, and 0.856). In addition, the OS of low-risk (total score ≤ 142.65) versus high-risk (total score > 142.65) patients varied significantly in both training group (P < 0.0001) and external validation cohort (P = 0.0012).

**Conclusions:**

According to this study, baseline biomarkers are associated with response to immunotherapy and prognosis among patients with metastatic melanoma. Treatment regimens can be tailor-made on the basis of these biomarkers.

## Introduction

As oncology advances, immunotherapy targeting immune checkpoints are gaining popularity, such as cytotoxic T lymphocyte antigen 4 (CTLA-4) and programmed cell death-1 (PD-1). Because the immune checkpoint inhibitors (ICIs) have emerged, they have rapidly been integrated into many cancer treatment regimens in the past decade. As the leading cause of skin cancer–related mortality, melanoma has the capacity to develop distant metastases (1). Despite the absence of curative options for advanced melanoma, the advent of immunotherapy dramatically improves the prognoses (2). In spite of their impressive effects on malignancies, there were still a majority of patients who were not able to benefit from the anti–PD-1/programmed death ligand 1 (PD-L1) monotherapy, and these treatments also induce a variety of immune-related adverse events (irAEs), some of which can be fatal (3–7). According to randomized clinical trials, the occurrence of endocrine irAEs during anti–PD-1 monotherapy ranged from 3.8% to 20.8%, whereas anti–PD-1 + anti–CTLA-4 combination treatment regimen has been associated with 14.4% to 34% (8). During anti–PD-1 immunotherapy, thyroid toxicity accounts for 5% to 15% of all irAEs (9, 10). Most commonly, symptoms of thyroid toxicity include transient thyrotoxicosis and hypothyroidism, similar to classical thyroiditis, although its precise cause remains unknown (11, 12). Previously, thyroid toxicity during immunotherapy may be linked with improved overall survival (OS); however, with small sample sizes, these studies may be limited, including patients with renal cell carcinoma and non–small cell lung cancer (NSCLC), and progression-free survival (PFS) showed inconsistent effects (13–15). Lactate dehydrogenase (LDH) is a vital physiological enzyme involved in enhanced aerobic glycolysis, catalyzing pyruvate’s reversible transformation to lactate. In addition, lactate accumulation reduced CD8^+^ T cell and natural killer cell survival and cytolytic capacity, promoting tumor immune escape (16). Numerous studies suggested that patients with cancer with a high level of LDH may lead to poor prognosis in multiple cancer types due to the antagonism of anti–PD-1/PD-L1 antibodies, and LDH blockade improves the effectiveness of anti–PD-1 therapy (17–19). There is an ongoing debate regarding whether LDH can be used to assess the prognosis of patients with melanoma treated with anti–PD-1/PD-L1 antibodies, because some evidence suggest that OS/PFS and pretreatment LDH have no significant correlation (20–22).

Currently, oncologic outcome nomograms can be used to evaluate risk by considering important clinical and pathological factors (23, 24). The creation of predictive nomograms may help both patients and physicians in making better management decisions. In addition to this, several types of cancer have shown that nomograms are more accurate than traditional TNM classifications (25, 26). However, nomograms based on Chinese population cohort to predict prognosis and guide immunotherapy in metastatic melanoma are rare.

Therefore, we hypothesized that the baseline levels of thyroid antibodies, thyroid status, and serum LDH and M stage before immunotherapy might predict treatment outcomes in patients with metastatic melanoma. In present study, on the basis of a large prospective cohort of Chinese patients with metastatic melanoma, we explored the correlation between several baseline factors and prognosis. Furthermore, we constructed a risk score model and developed a nomogram in conjunction with clinical characteristics. Identifying effective biomarkers to predict response and prognosis after immunotherapy may benefit in guiding treatment regimen.

## Materials and methods

### Patients and samples

Study approval was granted by the Ethics Committee of Sun Yat-sen University Cancer Center. We retrospectively collected 165 patients diagnosed with metastatic melanoma receiving anti–PD-1 treatment between March 2015 and December 2019 at Sun Yat-sen University Cancer Center. The inclusion criteria are as follows: (a) patients diagnosed with advanced melanoma, (b) patients who underwent anti–PD-1 treatment, (c) measurability of tumor lesions/lymph nodes, and (d) availability of clinical data. The exclusion criteria are as follows: (a) preoperative therapy (neoadjuvant radiotherapy or chemotherapy), (b) patients with other types of malignancies, (c) missing essential histopathological results, (d) missing essential imaging data to evaluate response, and (e) incomplete information.

We enrolled another 66 patients with metastatic melanoma who underwent anti–PD-1 antibody treatment between March 2017 and December 2021 as an external validation cohort that is also from the Sun Yat-sen University Cancer Center.

Serum was collected at baseline to determine free T3 (FT3; reference range: 2.80–7.10 pmol/L), free T4 (FT4; reference range: 12.00–22.00 pmol/L), thyroid-stimulating hormone (TSH; reference range: 0.27–4.20 uIU/ml), thyroglobulin (TG; reference range: 3.5–77.00 ng/ml), thyroid peroxidase antibody (A-TPO; reference range: 0–35.00 U/ml), and LDH (reference range: 120.00–250.00 U/L) levels.

### Follow-up

After anti–PD-1 treatment, we maintain a regular contact with patients through outpatient reexaminations, telephone calls, and hospital medical records at least until 5 years after the treatment or mortality. Patients with incomplete follow-up were censored. In this study, a follow-up is due by 28 February 2022. On the basis of the standard Response Evaluation Criteria In Solid Tumors (RECIST) version 1.1 (**Supplementary Table 1**) (27), OS is defined as the period between the start of therapy and death, whereas PFS is calculated until the tumor progresses or death. In melanoma, non-PD, composed of complete response (CR), partial response (PR), and simple disease (SD), which we always equate as “disease control rate”, predicts survival better than response alone.

### Statistical analysis

All data analysis was performed using SPSS 25.0 software (IBM, USA), GraphPad Prism 9 (GraphPad Software, Inc., USA), and R software 4.1.0 (https://www.r-project.org/). It is worth noting that we use the median as the cutoff value of thyroid function grouping. Chi-square test was used to analyze the correlation between clinical baseline characteristics and short-term efficacy of the PD-1 antibody. Survival analysis was performed by the Kaplan-Meier (K-M) method and tested by the log rank test. Univariate and multivariate analyses were performed by Cox regression model to evaluate the correlations between prognostic factors and OS, and the hazard ratios (HRs) and corresponding 95% confidence intervals (CIs) were shown. All statistical tests were two-sided, and P < 0.05 was supposed to be statistically significant.

### The nomogram establishing

Univariate and multivariate Cox professional hazard models were used to determine the potential important prognostic factors of the entire cohort. Multivariate Cox regression analysis includes variables with P < 0.05 in univariate analysis. If significant effects were observed in Cox model (P < 0.05), then they were determined as prognostic factors independently. Finally, serum A-TPO, FT3, and LDH levels and M stage were confirmed as the independent prognostic factors. In addition, on the basis of Cox professional hazards models, nomogram models were constructed in R using the “rms” package and were shown by the “ggplot2” package. 

### Discrimination and calibration of the nomogram

The nomogram was internally and externally validated, and the model discrimination and calibration were evaluated. The discrimination accuracy of the nomogram was estimated using the area under the curve (AUC). The AUC values range from 0.5 to 1. In case the AUC value is equal to 0.5, the nomogram has no ability to discriminate. On the contrary, if the AUC value is 1, then the model’s ability of stratifying patients into different prognosis groups is perfect. The calibration was evaluated through the calibration curve, which is a chart showing the correlation between the prediction probability and the observed result frequency. Standard curves are straight lines with slope 1 that pass through the origin of the coordinate axis. Nomograms are more accurate if the calibration curve is closer to the standard curve.

### Stratification of risk groups based on nomograms

On the basis of the nomogram, we calculated each patient’s sum score by the “nomogramFormula” R package. Subsequently, the “surv_cutpoint” function in the “survminer” R package generated the cutoff points for stratifying the risk into low and high, and then, we carried out the survival analysis of the subgroup by the “survival” R package.

## Results

### Patient characteristics

Baseline characteristics of patients (n = 165) are shown in **Table 1**. In total, 165 patients with diagnosed melanoma were included in the study. In addition, 80 female patients (48.5%) and 85 male patients (51.5%) were included in the sample. One-hundred eleven of the 165 patients (67.2%) were younger than 60 years, and 54 (32.8%) were older than 60 years. According to the baseline LDH level, patients were stratified into two groups: 103 patients (62.4%) had normal LDH (LDH = 0), and 62 patients (37.6%) had increased LDH (LDH = 1). In addition, there are 42 (25.5%) patients carried a V-raf murine sarcoma viral oncogene homolog B1 (BRAF) mutation, and 123 (74.5%) patients were wild type. In patients with distant metastases (M1A, n = 42; M1B, n = 30; M1C, n = 72; M1D, n = 21), we delineate the M1 stage into two M categories: The first is defined as [M1A + M1B] and the second is defined as [M1C + M1D]. According to the indices of the baseline thyroid functions, each subgroup was divided into the low (below the median value) and high (above or equal to the median value) groups (cutoff values of each subgroup: baseline FT3, 4.54 pmol/L; baseline FT4, 16.18 pmol/L; baseline TSH, 1.66 uIU/ml; baseline A-TPO: 13.09 U/ml; baseline TG, 9.19 ng/ml).

**Table 1:** Patient baseline characteristics.

		N	Proportion
Age(years)	<60	111	67.2%
	≥60	54	32.8%
Sex	Male	85	51.5%
	Female	80	48.5%
LDH	0	103	62.4%
	1	62	37.6%
M stage	M1A	42	25.5%
	M1B	30	18.2%
	M1C	72	43.6%
	M1D	21	12.7%
BRAF mutation	0	123	74.5%
	1	42	25.5%
Baseline			
FT3 (pmol/L)	Low (<4.535)	83	50.3%
FT4 (pmol/L)	High (≥4.535)	82	49.7%
	Low (<16.185)	83	50.3%
TSH (uIU/ml)	High (≥16.185)	82	49.7%
	Low (<1.665)	83	50.3%
A-TPO (U/ml)	High (≥1.665)	82	49.7%
	Low (<13.09)	82	49.7%
	High (≥13.09)	83	50.3%
TG (ng/ml)	Low (<9.19)	82	49.7%
	High (≥9.19)	83	50.3%

A-TPO, thyroid peroxidase antibody; FT3, free T3; FT4, free T4; LDH, lactate dehydrogenase; TG, thyroglobulin; TSH, thyroid-stimulating hormone; BRAF, V-raf murine sarcoma viral oncogene homolog B1.

### The association of short-term efficacy of PD-1 blockade therapy with several baseline predictor variables in melanoma

According to whether disease had progressed, patients were stratified into two groups: progression (PD) and non-progression (CR + PR + SD) groups. Clear correlation between baseline FT4 (P = 0.001) or M stage (P = 0.015) and short-term tumor response was evident, suggesting that blocking PD-1 with therapeutic antibodies would be useful if patients had a high-level baseline FT4 or earlier M stage. In addition, there was no obvious correlation between a short-term efficacy of PD-1 antibodies and age, sex, LDH levels, BRAF mutation, baseline FT3, baseline TSH, baseline A-TPO, or baseline TG (**Table 2**).

**Table 2:** The association of efficacy of PD-1 blockade therapy with several baseline predictor variables.

Variable	Effect		P-value
	DCR	PD	
Age			0.159
<60 (111)	54 (48.6%)	57 (51.4%)	
≥60 (54)	20 (37%)	34 (63%)
Sex			0.224
Male (85)	42 (49.4%)	43 (50.6%)	
Female (80)	32 (40%)	48 (60%)
LDH			0.061
0 (103)	52 (50.5%)	51 (49.5%)	
1 (62)	22 (35.5%)	40 (64.5%)	
Baseline			
FT3			0.102
Low (83)	32 (38.6%)	51 (61.4%)	
High (82)	42 (51.2%)	40 (48.8%)	
FT4			0.001
Low (83)	27 (32.5%)	56 (67.5%)	
High (82)	47 (57.3%)	35 (42.7%)	
TSH			0.486
Low (83)	35 (42.2%)	48 (57.8%)	
High (82)	39 (47.6%)	43 (52.4%)	
A-TPO			0.578
Low (82)	35 (42.7%)	47 (57.3%)	
High (83)	39 (47.9%)	44 (53.0%)	
TG			0.808
Low (83)	36 (43.9%)	46 (55.4%)	
High (82)	38 (45.8%)	45 (54.9%)	
M stage			0.015
0 (72)	40 (55.6%)	32 (44.4%)	
1 (93)	34 (36.6%)	59 (63.4%)	

A-TPO, thyroid peroxidase antibody; FT3, free T3; FT4, free T4; LDH, lactate dehydrogenase; TG, thyroglobulin; TSH, thyroid-stimulating hormone; PD-1, programmed cell death-1; DCR, Disease Control Rate; PD, progressive disease.

### Analysis of influencing factors of overall survival of Chinese patients with metastatic melanoma

**Figure 1:**
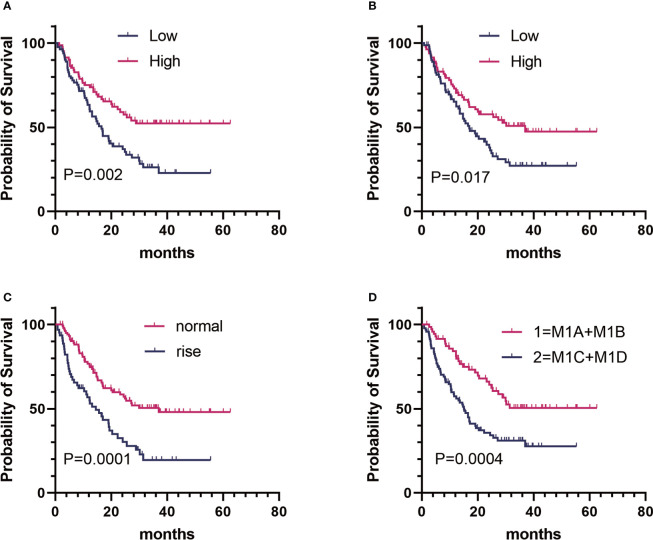
Kaplan–Meier estimates of overall survival (OS) of patients with metastatic melanoma in different groups. **(A)** Baseline FT3:OS curve of patients in the high group (n = 82) versus the low group (n = 83). **(B)** baseline A-TPO : OS curve of patients in the high group (n = 83) versus the low group (n = 82). **(C)** LDH level:OS curve of patients in the normal group (n = 103) versus the rise group (n = 62). **(D)** M stage:OS curve of patients in group 1 (n = 72) versus group 2 (n = 93).

On the basis of the univariate analysis, baseline FT3 (HR, 0.519; CI, 0.339–0.796; P = 0.002), baseline FT4 (HR, 0.625; CI, 0.410–0.950; P = 0.026), baseline A-TPO (HR, 0.601; CI, 0.394–0.917; P = 0.017), and LDH (HR, 2.220; CI, 1.461–3.374; P = 0.0001) levels and M stage (HR, 2.170; CI, 1.395–3.375; P = 0.0004) were significantly associated with OS (**Figure 1**, **Table 3**). In contrast, age, sex, baseline TSH, baseline TG, and BRAF mutation did not influence OS (P > 0.05). A multivariate Cox analysis incorporating meaningful univariate analysis variables showed that baseline FT3 (HR, 0.554; 95% CI, 0.355–0.865; P = 0.009), baseline A-TPO (HR, 0.567; 95% CI, 0.381–0.904; P = 0.016), and LDH (HR, 1.738; 95% CI, 1.121–2.693; P = 0.013) levels and M stage (HR, 2.156; 95% CI, 1.361–3.414; P < 0.001) independently predicted OS in patients with melanoma. In addition, it was confirmed in this analysis that baseline FT4 does not have an independent prognostic value for melanoma. **Tables 3**, **4** show results for univariate and multivariate Cox regression models.

**Table 3:** Univariate Cox regression analysis of influencing factors of overall survival (OS).

Variable	Median OS	Univariate		
	(months)	P-value	HR	95% CI
Total	22.3			
sex		0.172	0.747	0.490–1.138
Male	20.4			
Female	30.2			
Age		0.136	1.380	0.901–2.112
<60	27.1			
≥60	19.4			
Baseline				
FT3		0.002	0.519	0.339–0.796
Low	16.5			
High	>30			
FT4		0.026	0.625	0.410–0.950
Low	17.0			
HIGH	30.0			
TSH		0.283	1.255	0.827–1.904
Low	25.6			
High	20.4			
A-TPO		0.017	0.601	0.394–0.917
Low	17.0			
High	37.0			
TG		0.134	0.727	0.479–1.103
Low	19.0			
High	27.4			
M stage		<0.001	2.170	1.395–3.375
1 (M1A + M1B)	>30			
2 (M1C + M1D)	14.5			
LDH		<0.001	2.220	1.461–3.374
0	>30			
1	15.1			
BRAF mutation		0.191	0.718	0.436–1.183
None	19.4			
Mutation	30.0			

A-TPO, thyroid peroxidase antibody; CI, confidence interval; FT3, free T3; FT4, free T4; HR, hazard ratio; LDH, lactate dehydrogenase; TG, thyroglobulin; TSH, thyroid-stimulating hormone; BRAF, V-raf murine sarcoma viral oncogene homolog B1.

**Table 4:** Multivariate Cox regression analysis of influencing factors of overall survival (OS).

Variable	Median OS	Multivariate		
	(months)	HR	95% CI	P-value
Total	22.3			
				
Baseline				
FT3		0.554	0.355–0.865	0.009
Low	16.5			
High	>30			
FT4		0.791	0.514–1.217	0.286
Low	17.0			
High	30.0			
A-TPO		0.567	0.381–0.904	0.016
Low	17.0			
High	37.0			
M stage		2.156	1.361–3.414	0.001
1 (M1A + M1B)	>30			
2 (M1C + M1D)	14.5			
LDH		1.738	1.121–2.693	0.013
0	>30			
1	15.1			

A-TPO, thyroid peroxidase antibody; CI, confidence interval; FT3, free T3; FT4, free T4; HR, hazard ratio; LDH, lactate dehydrogenase.

### Prognostic nomogram for overall survival of Chinese patients with metastatic melanoma treated with anti–PD-1 antibodies 

**Figure 2:**
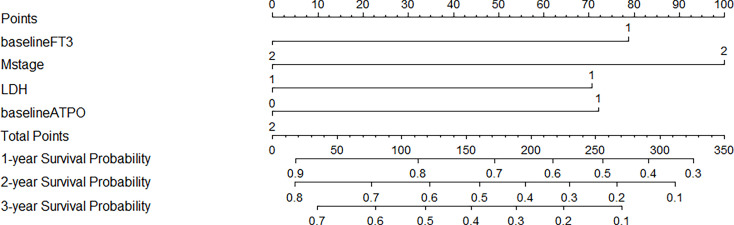
Prognostic Nomograms of 1-, 2-, and 3-year OS for patients with metastatic melanoma. For every patient in the nomogram, four lines are drawn upward to calculate the points received from the four predictors. The sum of the points is located on the “Total Points” axis. In addition, the possibility of 1-, 2-, and 3-year OS is determined by drawing a downward line.

The prediction model is presented in the form of nomogram (**Figure 2**). The variables included in the model were baseline FT3 (1 = low, 2 = high), baseline A-TPO (1 = low, 2 = high), M stage (1 = M1A or M1B, 2 = M1C or M1D), and LDH (normal = 0, elevated = 1) levels. A score is assigned to each independent predictor by writing a line pointing directly to the axis of score. The total score is obtained by adding up the scores of all related factors, and the corresponding predicted survival probability can be obtained by making a vertical line from the total score axis to intersect the survival probability axis of 1, 2, and 3 years. For example, a patient with stage M1C melanoma (100 points) with normal LDH level (0 points), whose baseline FT3 is 2.25 pmol/L (78 points) and baseline A-TPO is 17.24 U/ml (0 points), has a sum score equal to 178, which corresponds to the foreseen survival probability of 1, 2, and 3 years of 69%, 44%, and 33%, respectively (**Supplementary Table 2**).

### Calibration and verification of nomogram

**Figure 3:**
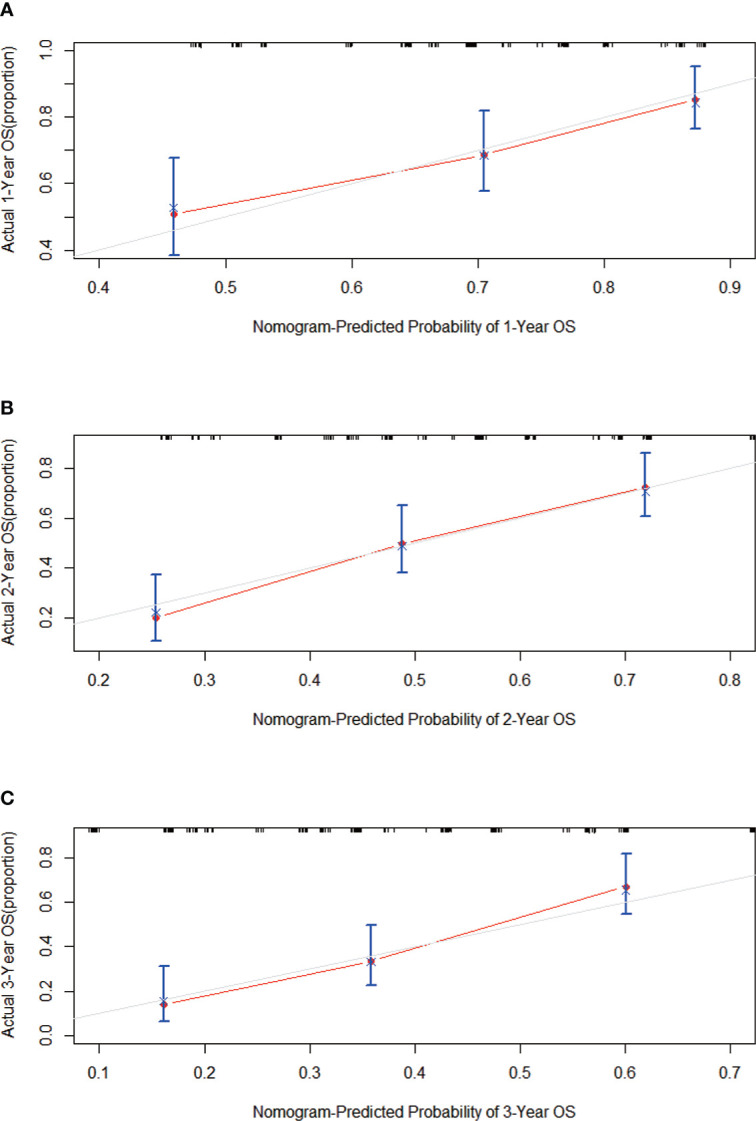
Calibration curves predicting the **(A)** 1-, **(B)** 2-, and **(C)** 3-year OS of patients in the training cohort. On the x-axis are the predicted survival probabilities, and on the y-axis are the actual survival probabilities. There is an agreement between the prediction and reality based on the 45° line (gray line).

**Figure 4:**
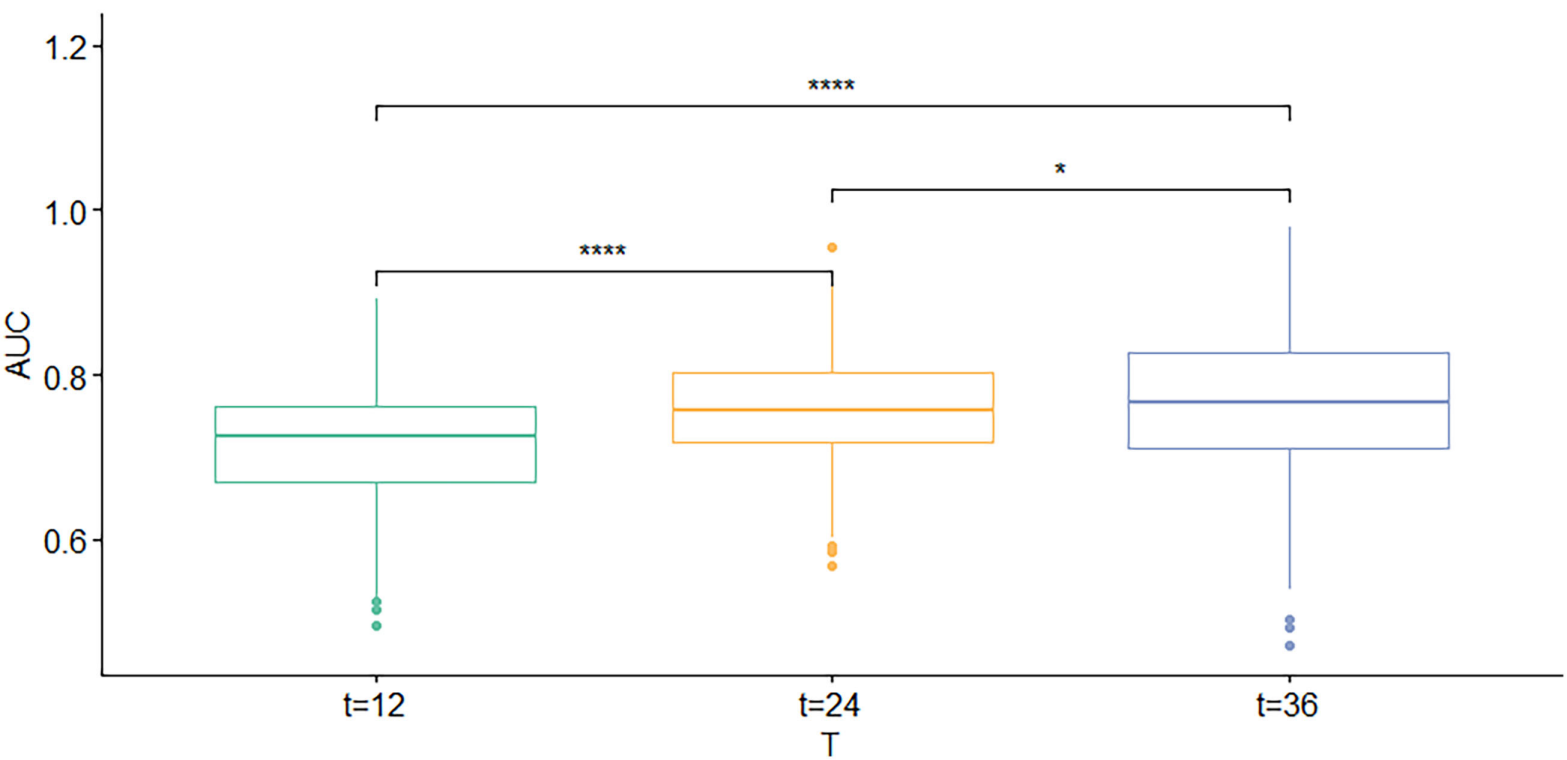
Box map: AUC mean value of 1-, 2-, and 3-year survival probability.

**Figure 5:**
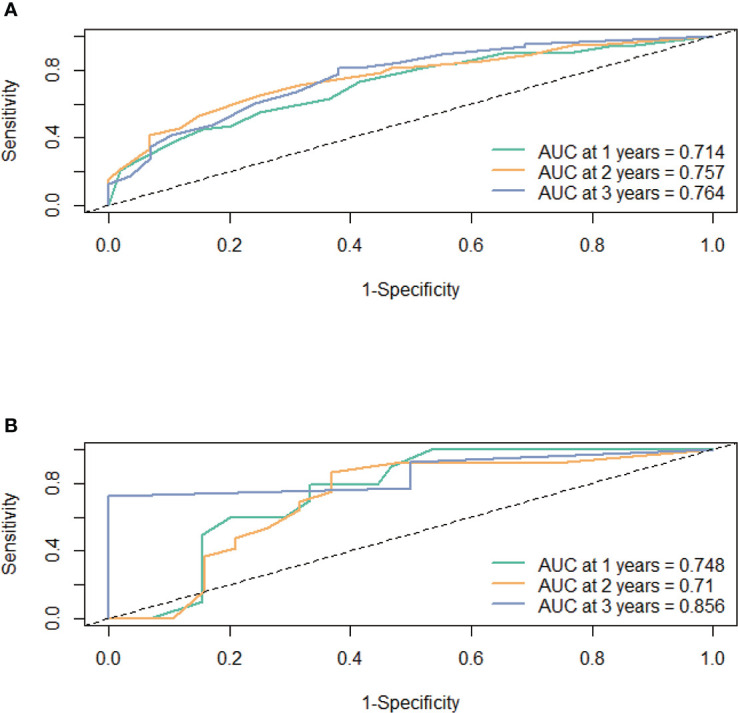
The prognostic accuracy of the nomogram was estimated by using ROC curves and AUCs at 1, 2, and 3 years in the training cohort **(A)** and the external validation cohort **(B)**.

First, the calibration curve shows that, in the training cohort, the actual observations are in excellent agreement with the prediction results of the nomogram (**Figure 3**). Second, we carried out internal verification. The internal verification is carried out by randomly segmenting the data set at 7:3 and repeating it 500 times, calculating the AUC value, and counting its mean value, respectively. The 1-year survival probability AUC mean is 0.7171963, the 2-year survival probability mean value is 0.756549, and the 3-year survival probability mean value is 0.7651486 (**Figure 4**), which showed a good discrimination ability in the training cohort made up of 165 Chinese patients with metastatic melanoma. Last, prognostic accuracy and clinical applicability of nomogram were evaluated using ROC curves and AUC values of 1-, 2-, and 3-year survival probability in the training cohort and the external verification cohort. The AUC values in the ROC curve analysis showed a good accuracy (the AUC values of 1-, 2-, and 3-year survival probability in the training cohort were 0.714, 0.757, and 0.764 and in the external verification cohort were 0.748, 0.710, and 0.856, respectively) (**Figure 5**).

### Risk assessment capabilities of the nomogram

**Figure 6:**
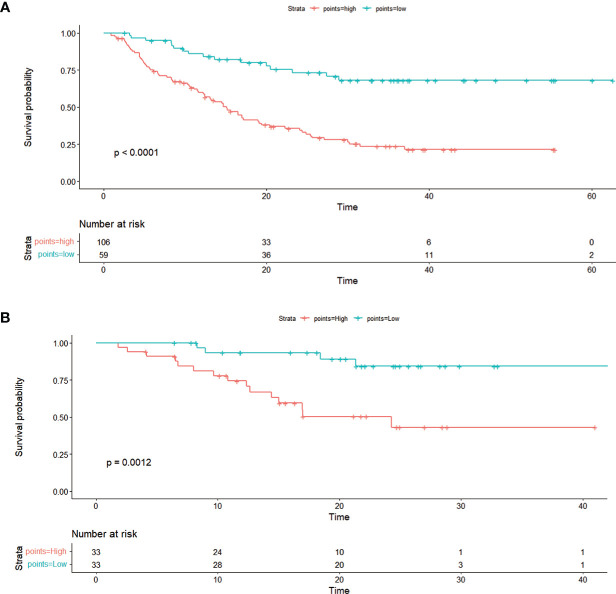
The Kaplan–Meier curve shows OS with risk stratification in the training cohort **(A)** and in the external validation cohort **(B)**.

On the basis of the nomogram, 142.65 was the cutoff value for the sum score (**Supplementary Figure S1**). We divided all patients into two risk groups based on the cutoff value (142.65): high-risk group (>142.65) and low-risk group (≤142.65). There was a remarkable difference in OS between individuals who were at low risk and those who were at high risk (P < 0.0001). When the unchanged cutoff value was applied to the external validation cohort, it was also possible to discriminate between high- and low-risk OS in Kaplan-Meier (K-M) curves. Observations show that the OS of low-risk group is overwhelmingly superior to that of the high-risk group OS (P = 0.0012) (**Figure 6**).

## Discussion

Melanoma is one of the most aggressive forms of skin cancer, accounting for 5% of all cases, but 80% of mortality is related to it (28). Therefore, immunotherapy is administered to patients with metastatic melanoma as an adjuvant treatment (29). Nevertheless, not all patients benefit from immunotherapy, so new methods that predict treatment response are needed. Several studies have shown that thyroid antibodies, thyroid status, serum LDH, and M stage are all predictors of immunotherapy response, but they each have their limitations (15, 30). Thus, further research into the association may be of value in developing a combined biomarker model that can better predict the response in advanced melanoma.

Our study explored the relationship between various baseline clinicopathological factors before treatment and the short-term efficacy of PD-1 antibody. Chi-square test showed that PD-1 antibody was more effective in patients with melanoma with higher level baseline FT4 or earlier M stage. Furthermore, in **Table 2**, we can see that lower baseline FT3 is associated with a worse efficacy [more patients with progressive disease (PD)], which is consistent with the results of Cox that lower baseline FT3 levels are correlated with worse prognosis (shorter OS in the low-level baseline FT3 group). The reason why the statistical value is meaningless (P > 0.05) should be that the sample size is not big enough.

It has been proven that the comprehensive analysis of baseline clinicopathological factors by nomogram can accurately predict the clinical results of metastatic melanoma checkpoint inhibitor immunotherapy (31). However, it is still an unsolved challenge to use clinical indicators to stratify the prognosis of metastatic melanoma treated with PD-1 antibody and predict its oncological outcome in the Chinese population. The research described here is the first to develop a clinically useful nomogram for accurately predicting the outcomes of checkpoint inhibitor immunotherapy in metastatic melanoma in Chinese patients, based on a comprehensive analysis of a variety of baseline clinicopathological variables. In this study, the patient cohort was originated from the Sun Yat-sen University Cancer Center (Guangzhou, China), one of the largest cancer hospitals in China. It appears that the sample enrolled in this cohort is both generalizable and representative of Chinese patients with melanoma. Our study explored the relationship between the baseline pretreatment clinicopathologic factors and the prognosis of PD-1 antibody therapy. It may assist clinicians to identify patients with poor prognosis in PD-1 antibody therapy, who may be in need of novel clinical trials options at the outset (i.e., first line).

There have been several clinical (e.g., presence/absence of liver and brain metastases) (30, 32) and hematologic factors (e.g., LDH) (33) that have been described to be linked to ICI response or resistance (34). In the majority of the cases, these factors, however, have not been included in multivariable models. In this study, we verified that baseline FT3, baseline A-TPO, LDH, and M stage (presence/absence of liver and brain metastases) were the independent prognostic factors of OS in patients with metastatic melanoma treated with PD-1 antibody, through the univariate and multivariate analyses. LDH, a factor of the melanoma American Joint Committee on Cancer (AJCC) staging, is a prognostic factor for other types of cancer. In addition, LDH has also proved to be a predictive marker, and a higher level of LDH is related with shorter PFS and OS in immunotherapy (35, 36). In addition, studies have shown that baseline FT3 levels are negatively correlated with cancer mortality (37, 38). Patients with baseline A-TPO level above the median had a higher OS (15), and the presence of liver or brain metastases is associated with shorter OS (31). The results of this study support these conclusions.

A nomogram was developed on the basis of the risk score model and clinical features to predict the survival possibility in metastatic melanoma. The nomogram was established according to four independent risk factors, and the patients were divided into high-risk group and low-risk group by the ROC curve analysis. It is encouraging to note that low-risk patients had a significantly better OS than high-risk patients. There is an excellent agreement between the actual observations and the predictions of the nomogram, as shown by the calibration curve. In China, we lack indicators for prognostic assessment and risk subgroup stratification of patients with metastatic melanoma treated with PD-1 antibody. Therefore, the line chart can help Chinese doctors in using this Chinese doctors’ scoring system to predict the individual survival of patients with metastatic melanoma. Aside from that, clinical studies can benefit from the scoring system because the system can provide stratified information for patients to decrease sample selection deviations.

It is important to note, however, that the current study still has some limitations that must be considered. Statistically valid risk score models and nomograms must be validated in larger clinical cohorts to determine the impact of thyroid antibodies, thyroid status, serum LDH, and M stage on response of immunotherapy and prognosis. In addition, it is necessary to validate our nomogram with a different experimental cohort. However, because of the limitation of data acquisition, we cannot do this for the time being. Moreover, more research studies are required to dynamically detect the level of serum thyroid hormone thyroid antibodies and LDH during the process of anti–PD-1 treatment and to verify the relationship between these factors and prognosis. Overt thyroid toxicity during treatment significantly prolonged OS and PFS in patients with metastatic melanoma, NSCLC, and renal cell carcinoma (RCC) receiving anti–PD-1. Previous studies in recent years assumed that these patients with higher levels of autoimmunity may benefit from anti-cancer treatment *via* autoimmune pathways because they are more susceptible to autoimmunity, which is supported by numerous studies indicating a positive correlation between irAEs and improved response and survival rates in patients treated with anti–PD-1 (39–44). There were, however, a number of limitations to most of these studies, including their retrospective design and/or small sample size; while comparing with published associations with other irAEs, we found that thyroid toxicity had much larger effect sizes on OS in our current retrospective study (39–44). In addition, the number of samples in this study is limited. Hence, to validate the model, additional external cohorts will be required. As a result, risk factors play an essential role in predicting clinical response and prognosis in patients with metastatic melanoma after anti–PD-1 treatment and are in need of further validation and updating in the future.

## Conclusions

In conclusion, this study construct a nomogram based on the baseline levels of thyroid antibodies, thyroid status, serum LDH, and M stage before anti–PD-1, showing that the factors are strong predictive markers for response and prognosis to anti–PD-1 treatment in metastatic melanoma. This parameter may serve as a novel effective marker to predict response and prognosis, therefore assisting in treatment regimen selection.

## Data availability statement

The raw data supporting the conclusions of this article will be made available by the authors, without undue reservation.

## Ethics statement

The studies involving human participants were reviewed and approved by Ethics Committee of Sun Yat-sen University Cancer Center. The patients/participants provided their written informed consent to participate in this study. Written informed consent was obtained from the individual(s) for the publication of any potentially identifiable images or data included in this article.

## Author contributions

All authors participated in the present study. YD, XZ, and YT contributed to the conception and design. JZ, DL, SX, XW, JL, XY, and ML conducted the collection of data. JZ, DL, and ZW analyzed and interpreted the data. SX and XD visualized the result. JZ and SX drafted the manuscript. All authors read and approved the manuscript.
