# Supplementary figures and images for: Nomogram for predicting prognosis of patients with metastatic melanoma after immunotherapy: A Chinese population–based analysis
Source: Front Immunol. 2022 Dec 22;13:1083840. doi: 10.3389/fimmu.2022.1083840 (PMC9815596; doi:10.3389/fimmu.2022.1083840)

**Supplementary Fig. S1 The optimal cut-off value determined by the sum-score was 142.65.**

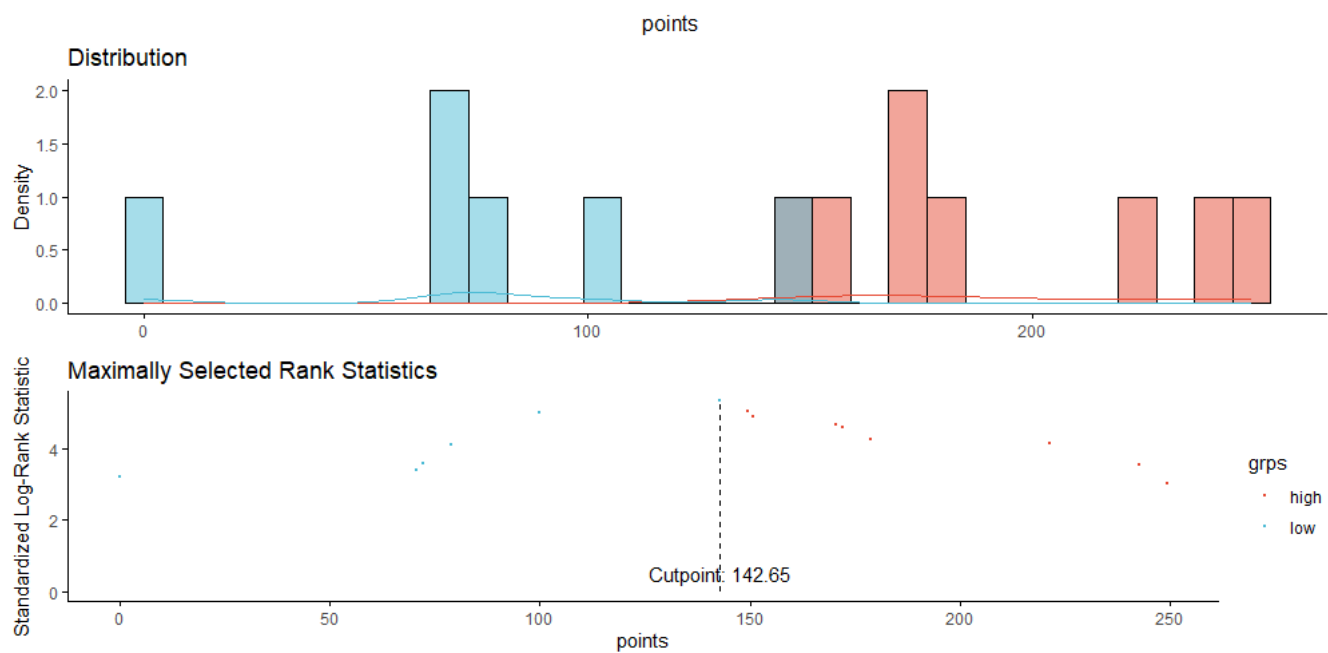

Supplement: Supplementary file 1 [file Image_1.pdf]
