# Supplementary material for: Nomogram for predicting prognosis of patients with metastatic melanoma after immunotherapy: A Chinese population–based analysis
Source: Front Immunol. 2022 Dec 22;13:1083840. doi: 10.3389/fimmu.2022.1083840 (PMC9815596; doi:10.3389/fimmu.2022.1083840)
Supplement: Supplementary file 2 [file DataSheet_1.pdf]

Supplementary Table 1 Response Evaluation Criteria In Solid Tumors (RECIST) Version 1.1

Complete Response (CR)	Disappearance of all target lesions. Any pathological lymph nodes (whether target or non-target) must have reduction in short axis to <10 mm.
Partial Response (PR)	At least a 30% decrease in the sum of diameters of target lesions, taking as reference the baseline sum diameters.
Stable Disease (SD)	Neither sufficient shrinkage to qualify for PR nor sufficient increase to qualify for PD, taking as reference the smallest sum diameters while on study.
Progressive Disease (PD)	At least a 20% increase in the sum of diameters of target lesions, taking as reference the smallest sum on study (this includes the baseline sum if that is the smallest on study). In addition to the relative increase of 20%, the sum must also demonstrate an absolute increase of at least 5 mm. And the appearance of one or more new lesions.
