# Supplementary material for: Nomogram for predicting prognosis of patients with metastatic melanoma after immunotherapy: A Chinese population–based analysis
Source: Front Immunol. 2022 Dec 22;13:1083840. doi: 10.3389/fimmu.2022.1083840 (PMC9815596; doi:10.3389/fimmu.2022.1083840)
Supplement: Supplementary file 3 [file DataSheet_2.pdf]

	Baseline FT3	Baseline A-TPO	LDH	M stage
Value	2.25pmol/L	17.24U/mL	normal	M1C
Group	1	2	0	2
Score	78	0	0	100
Total score	=78+0+0+100=178			
1-year survival probability	69%			
2-year survival probability	44%			
3-year survival probability	33%			
